# A11 NEXT STEPS IN MICRO-ELIMINATION: PEER POINT OF CARE HEPATITIS C TESTING IN VICTORIA, BRITISH COLUMBIA

**DOI:** 10.1093/jcag/gwac036.011

**Published:** 2023-03-07

**Authors:** T Barnett, M Selfridge, A Drost, K Guarasci, K Lundgren, C Fraser, F Boothman

**Affiliations:** 1 Cool Aid Community Health Centre; 2 Canadian Institute of Substance Use Research, University of Victoria, Victoria; 3 Department of Family Medicine, University of Victoria, Vancouver , Canada

## Abstract

**Background:**

Canada is currently on target to reach the 2030 WHO goal of hepatitis C virus (HCV) elimination. Continued high rates of treatment initiation are required to meet this goal. People who use drugs (PWUD), account for the majority of new HCV cases in BC and continue to have many barriers to accessing DAA therapies, despite demonstrated high SVR rates in clinical trials. Improved elimination efforts including innovative outreach testing and treatment with this population are essential. Novel models have proven successful to engage PWUD in HCV therapy with a simplified, task-shifted cascade of care. Peer-based testing and support models have been piloted in other communities and may help connect to marginalized populations. People with lived and living experience of HCV treatment and drug use (peers) are seen as trusted sources of knowledge. Peers can vouch for the efficacy, minimal side effects and ease of HCV treatment that now exist in the current DAA era.

**Purpose:**

The Peer HCV Point of Care (POC) testing project developed within our long standing nurse-led HCV treatment program seeks to determine whether an outreach peer model of HCV POC testing can be successful in Victoria, BC. The peer program focuses on finding populations who use drugs without regular access to primary care still living with HCV. This task shifting approach is the next phase of local micro-elimination efforts and has not been attempted previously.

**Method:**

Six peers have been trained to provide HCV POC antibody and dried blood spot RNA tests. Our goals are to pilot the program, learn from our experiences, specifically from the direct input of peers to develop effective and supportive testing and treatment strategies. Peers have worked with nursing and research staff in two-hour blocks and are paid $26 per hour for these shifts. They provide testing at local supportive housing, shelters, social service sites and special events. Each client tested is offered a $10 incentive. Our peers are able to offer both POC antibody testing and, for those who have been exposed to HCV (currently infected, treated or cleared), RNA testing by dried blood spot. Serology by nursing from our mobile outreach van is collected as needed.

**Result(s):**

Within the first 4 months of the project peers and staff tested 304 people: 251 people with HCV POC antibody tests (227 negative and 24 positive results), 41 people with HCV dried blood spot RNA tests and 28 with nurse RNA serology. To date 12 people tested RNA+ (11 with previously unknown HCV have active RNA that require treatment), 7 people have been started on treatment.

**Conclusion(s):**

This innovative and novel approach to HCV therapy in PWUD was able to successfully use a peer-based approach to find people with limited connection to primary health care to test and treat HCV. We still have much to learn from the valuable knowledge, established relationships and novel perspectives of peers in our efforts to reduce barriers and reach PWUD and others who remain untreated.

**Please acknowledge all funding agencies by checking the applicable boxes below:**

None

**Disclosure of Interest:**

T. Barnett Grant / Research support from: AbbVie, Gilead, Merck, M. Selfridge Grant / Research support from: Kirby Institute, AbbVie, Gilead, Merck, ViiV, A. Drost Grant / Research support from: Abbvie, Gilead, Merck, K. Guarasci Grant / Research support from: Gliead, Abbvie, Merck , K. Lundgren Grant / Research support from: Abbvie, Gilead, Merck , C. Fraser Grant / Research support from: Abbvie, Gilead, Merck, Kirby Institute, ViiV, F. Boothman : None Declared

